# Nimbolide, a neem limonoid inhibits Phosphatidyl Inositol-3 Kinase to activate Glycogen Synthase Kinase-3β in a hamster model of oral oncogenesis

**DOI:** 10.1038/srep22192

**Published:** 2016-02-23

**Authors:** Josephraj Sophia, Kranthi Kiran Kishore T., Jaganathan Kowshik, Rajakishore Mishra, Siddavaram Nagini

**Affiliations:** 1 Department of Biochemistry and Biotechnology, Faculty of Science, Annamalai University, Annamalainagar-608 002, Tamil Nadu, India; 2 Centre for Life Sciences, School of Natural Sciences, Central University of Jharkhand, Ratu-Lohardaga Road, Brambe, Ranchi 835205, Jharkhand, India

## Abstract

Glycogen synthase kinase-3β (GSK-3β), a serine/threonine kinase is frequently inactivated by the oncogenic signalling kinases PI3K/Akt and MAPK/ERK in diverse malignancies. The present study was designed to investigate GSK-3β signalling circuits in the 7,12-dimethylbenz[a]anthracene (DMBA)-induced hamster buccal pouch (HBP) carcinogenesis model and the therapeutic potential of the neem limonoid nimbolide. Inactivation of GSK-3β by phosphorylation at serine 9 and activation of PI3K/Akt, MAPK/ERK and β-catenin was associated with increased cell proliferation and apoptosis evasion during stepwise evolution of HBP carcinomas. Administration of nimbolide inhibited PI3K/Akt signalling with consequent activation of GSK-3β thereby inducing trafficking of β-catenin away from the nucleus and enhancing the expression of miR-126 and let-7. Molecular docking studies confirmed interaction of nimbolide with PI3K, Akt, ERK and GSK-3β. Furthermore, nimbolide attenuated cell proliferation and induced apoptosis as evidenced by increased p-cyclin D1^Thr286^ and pro-apoptotic proteins. The present study has unravelled aberrant phosphorylation as a key determinant for oncogenic signalling and acquisition of cancer hallmarks in the HBP model. The study has also provided mechanistic insights into the chemotherapeutic potential of nimbolide that may be a useful addition to the armamentarium of natural compounds targeting PI3K for oral cancer treatment.

Oral squamous cell carcinoma (OSCC) is one of the major global health problems with an annual estimated incidence of 300,000 newly diagnosed cases^1^. Tobacco consumption is recognized as the single most important risk factor accounting for 90% of oral cancer in India^2^. Despite advances in diagnosis and treatment, the 5-year survival rate of oral cancer has not improved significantly^3^. Novel molecularly targeted therapeutic strategies are therefore required to inhibit tumour development and progression. Kinases such as glycogen synthase kinase-3β (GSK-3β) that function as central links between key players regulating cell cycle progression, apoptosis, invasion and angiogenesis have emerged as promising molecular targets for oral cancer^4^,^5^,^6^.

GSK-3β, a serine/threonine kinase is involved in glycogen synthesis as well as in signalling pathways that regulate cell fate, protein synthesis, cell mobility, proliferation and survival^5^,^6^,^7^. The activity of GSK-3β that plays a central role in the Wnt/β-catenin pathway is regulated by site-specific phosphorylation of Ser9/Tyr216 residues. In the absence of Wnt signals, the transcription factor β-catenin maintained in a multiprotein complex with GSK-3β, axin, casein kinase (CK1), and adenomatous polyposis coli (APC), undergoes sequential phosphorylation by CK1 and GSK-3β followed by ubiquitination and proteasomal degradation. Binding of Wnt ligands to the frizzled receptor activates Wnt signalling leading to phosphorylation and inactivation of GSK-3β. This results in accumulation of stabilized cytosolic β-catenin which then translocates to the nucleus where it transactivates genes involved in cell cycle progression, invasion, metastasis, angiogenesis, and apoptosis^8^,^9^.

Several upstream kinases are involved in the inactivation of GSK-3β including phosphatidylinositol 3 kinase (PI3K/Akt), ribosomal S6 kinase (p90RSK), protein kinase A and C, and mitogen activated protein kinase (MAPK)^10^,^11^. GSK-3β is primarily inactivated by the PI3K/Akt and extracellular signal-regulated kinase (ERK)/MAPK pathways. PI3K recruits Akt to the plasma membrane which is then activated by phosphatidylinositol-dependent kinase (PDK) 1 and 2. Activated Akt, in turn, phosphorylates GSK-3β at Ser^9^, leading to activation of the Wnt signalling pathway^12^. Analysis of sequential changes in these pathways in an animal tumour model is of paramount importance to understand the role of GSK-3β and evolve therapeutic strategies for human OSCC.

The hamster buccal pouch (HBP) carcinogenesis model is one of the most well characterised animal systems to analyse the stepwise evolution of oral cancer and for chemointervention studies^13^. We have extensively used this model to test the chemopreventive and therapeutic potential of a wide range of phytochemicals derived from the diet and medicinal plants^14^,^15^,^16^,^17^. Nimbolide, a limonoid isolated from the leaves and flowers of the neem tree (*Azadirachta indica A.Juss*), has emerged as one of the most promising chemopreventive agents based on its potent inhibitory effects on the development of HBP carcinomas^17^,^18^,^19^. The antiproliferative and apoptosis inducing effects of nimbolide have been extensively documented in a panel of cancer cell lines^20^,^21^,^22^,^23^. The present study was designed to investigate the chemotherapeutic potential of nimbolide in the HBP model based on its modulatory effects on GSK-3β signalling circuits and the impact on key molecules involved in cell proliferation and apoptosis. In addition, we compared the effects of nimbolide with wortmannin (C_23_H_24_0_8_), a well-known potent and irreversible PI3K inhibitor^24^.

## Results

### Tumour incidence and sequential histopathological changes in the hamster buccal pouch

The tumour incidence, preneoplastic and neoplastic changes in the buccal pouch mucosa of hamsters painted with 7,12 dimethylbenz[a]anthracene (DMBA) is summarized in Supplementary Table S1. Erythroplakia with associated hyperplastic changes were observed in hamsters painted with DMBA for 4 weeks followed by small dysplastic nodules after 8 weeks, whereas well-defined exophytic tumours were seen after 12 weeks of DMBA painting. The buccal pouch epithelium of control animals was normal, intact, and continuous (Supplementary Fig. S1).

### Expression of GSK-3β and β-catenin during sequential progression of OSCC

**Figure 1:**
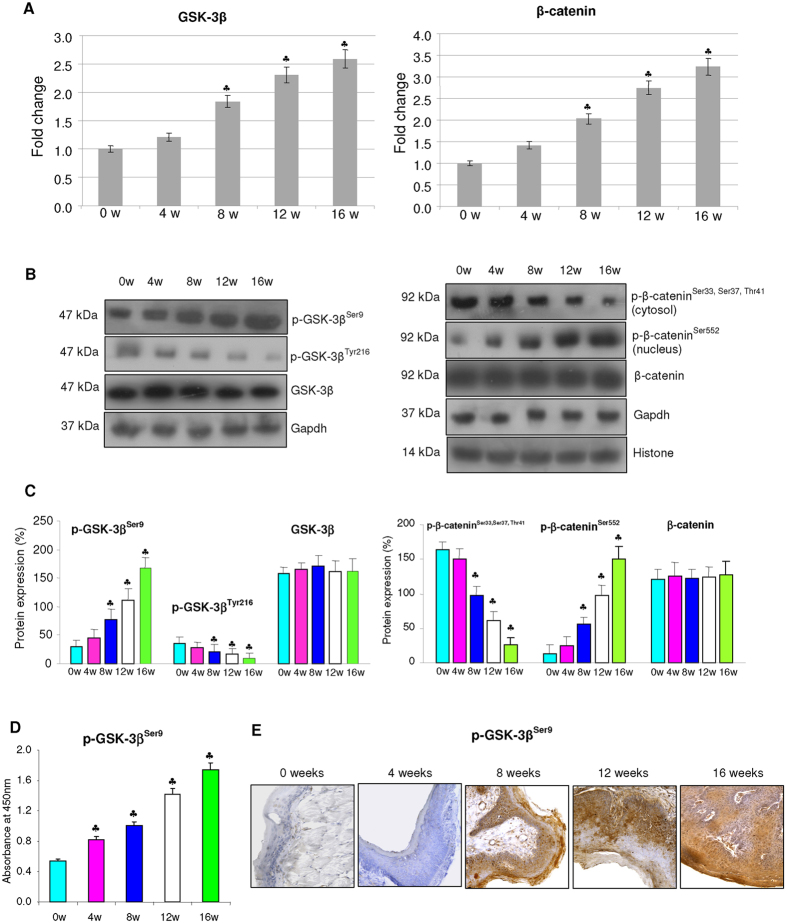
mRNA and protein expression of GSK-3β and β-catenin during the sequential progression of HBP carcinomas (mean ± SD; n = 3). (**A**) Transcript expression level of GSK-3β and β-catenin in control and DMBA painted hamster groups as determined by quantitative RT-PCR. The fold change in transcript expression for each gene was determined using the 2−ΔΔCt method. Data are the mean ± SD of three independent experiments. Statistical significance was determined by the Mann–Whitney test (p < 0.05). (**B**) Immunblots showing expression levels of GSK-3β and β-catenin in control and DMBA treated animals. Gapdh and histone H2B were used as loading control for cytosol and nuclear proteins respectively. (**C**) Background subtracted protein bands were quantified and normalized to Gapdh. Phosphorylated proteins are normalized by their unphosphorylated forms. Each bar represents the protein expression ± SD of three determinations.(**D**) p-GSK-3β^**Ser9**^ quantitated by ELISA showing a progressive increase fro 0 to 16 weeks of DMBA painting. (**E**) Representative immunostaining showing the expression of p-GSK3β^Ser9^ in various cellular compartments in control and experimental animals (×20). p < 0.05 versus control. The groups are indicated as 0w (0 weeks), 4w (4 weeks), 8w (8 weeks), 12w (12 weeks), 16w (16 weeks).

A sequential increase in the transcript and protein expression of p-GSK-3β^Ser9^ (inactive form) was associated with decreased expression of p-GSK-3β^Tyr216^ (active form) during progression of HBP carcinomas (Fig. 1). This was accompanied by a gradual increase in the expression of p-β-catenin^Ser552^ (active form) with a simultaneous downregulation of inactive p-β-catenin^Ser33, Ser37, Thr41^ in DMBA painted hamsters relative to control. GSK-3β inactivation was further confirmed by ELISA analysis.

### Expression of PI3K/Akt and ERK

**Figure 2:**
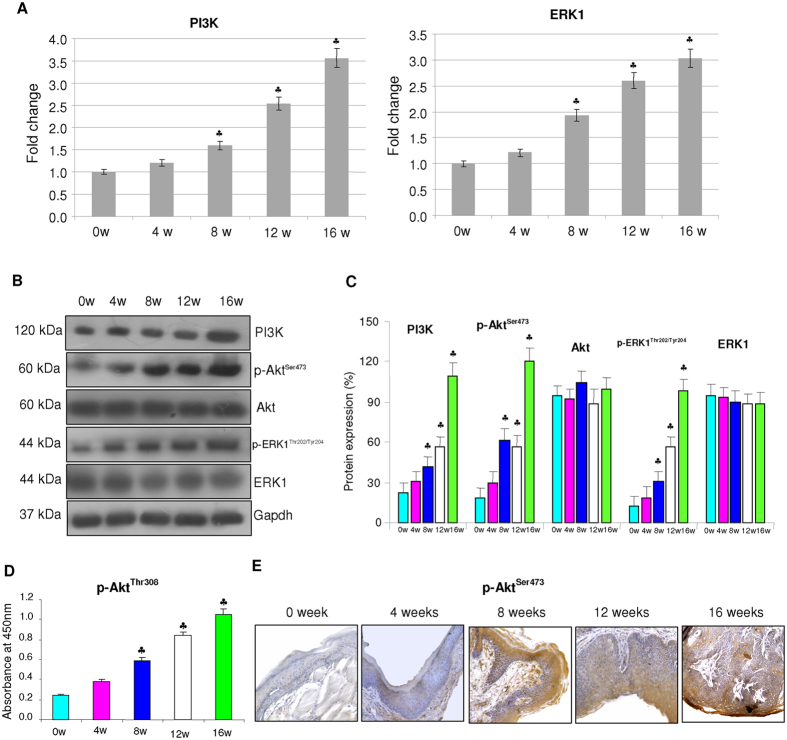
mRNA and protein expression of upstream kinases in the buccal pouch tissues during the stepwise progression of HBP carcinomas (mean ± SD; n = 3). (**A**) mRNA expression level of PI3K and ERK in control and DMBA painted animals as determined by kinetic PCR. The fold change in transcript expression for each gene was determined using the 2−ΔΔCt method. Data are the mean ± SD of three independent experiments. Statistical significance was determined by the Mann–Whitney test (p < 0.05). (**B**) Western blots showing overexpression of PI3K, p-Akt^Ser473^ and p-ERK1/2^Thr202/Tyr204^ in DMBA painted animals from 0 week to 16 weeks. Gapdh was used as loading control. Phosphorylated proteins are normalized by their unphosphorylated forms. (**C**) Background subtracted protein bands quantified and normalized to Gapdh. Phosphorylated proteins are normalized by their unphosphorylated forms. Each bar represents the protein expression ± SD of three determinations. (**D**) Total p-Akt^Thr308^ as determined by ELISA showing a sequential increase from 0 to 16 weeks. (**E**) Representative photomicrographs of immunohistochemical staining of pAkt^Ser473^ in control and experimental animals (×20). ^♣^p < 0.05 versus control.

We next analysed the expression of PI3K/Akt and ERK, primary upstream inhibitors of GSK-3β by quantitative RT-PCR and western blotting. Our results revealed a progressive increase in the expression of PI3K as well as phosphorylated Akt and ERK during the stepwise progression of SCC relative to control (Fig. 2).

### Effect of GSK-3β inactivation on cell proliferation and apoptosis

**Figure 3:**
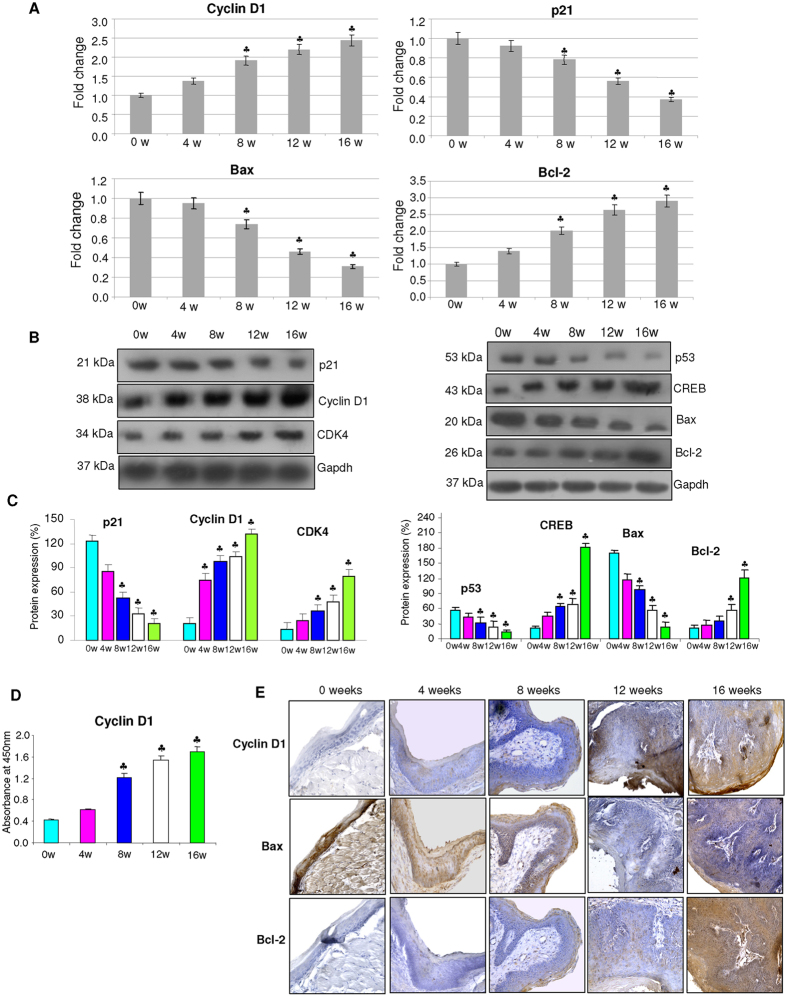
The expression of key molecules involved in cell cycle and apoptosis during the sequential progression of HBP carcinomas (mean ± SD; n = 3). (**A**) Quantitative RT-PCR analysis of Cyclin D1, p21, Bax and Bcl-2 in the buccal pouch tissues of control and DMBA painted hamsters determined by kinetic PCR. The fold change in transcript expression for each gene was determined using the 2−ΔΔCt method. Data are the mean ± SD of three independent experiments. Statistical significance was determined by the Mann–Whitney test (p < 0.05). (**B**) Western blots showing progressive increase in the expression of cyclins, anti-apoptotic proteins and decreased expression of p21 and pro-apoptotic proteins in DMBA painted hamsters from 0w to 16w. (**C**) Densitometric analysis representing expression of key proteins involved in cell proliferation and apoptosis for three independent experiments. (**D**) Levels of total cyclin D1 quantitated by ELISA showing a progressive increase from 0 to 16 weeks of DMBA painting.(**E**) Representative photomicrographs of immunohistochemical staining of Cyclin D1, Bax and Bcl-2 in control and DMBA painted animals (×40). ^♣^p < 0.05 versus control.

Since GSK-3β and activation of Wnt signalling regulate transcription of genes involved in cell proliferation and apoptosis, we analysed the expression of key molecules involved in these events (Fig. 3). We found a sequential increase in the expression of cyclin D1 and cyclin dependent kinase-4 (CDK4) with simultaneous significant downregulation of the cell cycle inhibitor p21 in hamsters painted with DMBA relative to control. The levels and expression of cyclin D1 was validated by ELISA and immunohistochemical analysis respectively. Transcript and protein expression analysis revealed a progressive shift to an anti-apoptotic phenotype. In particular, changes in the expression of p53 and cAMP response element-binding protein (CREB) that are recognised to regulate transcription of members of the Bcl-2 family, indicate apoptosis evasion in DMBA painted animals.

Pronounced changes in the expression of GSK-3β and β-catenin and the upstream signalling kinases that correlated with the appearance of dysplastic nodules in the HBP after 12 weeks of DMBA painting, prompted us to test the therapeutic efficacy of nimbolide at this time point. We used wortmannin, a known inhibitor of the PI3K/Akt pathway as a reference compound. Oral administration of nimbolide (100 μg/kg bw) and wortmannin (500 μg/kg bw) to DMBA painted hamsters for 4 weeks from the 12^th^ to the 16^th^ week, significantly reduced tumour burden. The tumour growth delay was significant in the group administered nimbolide (Supplementary Table S2). It is noteworthy that nimbolide exhibited significant chemotherapeutic potential at a concentration five times lower than that used for wortmannin.

### Nimbolide increases GSK-3β activity by modulating the expression of upstream kinases and microRNAs

**Figure 4:**
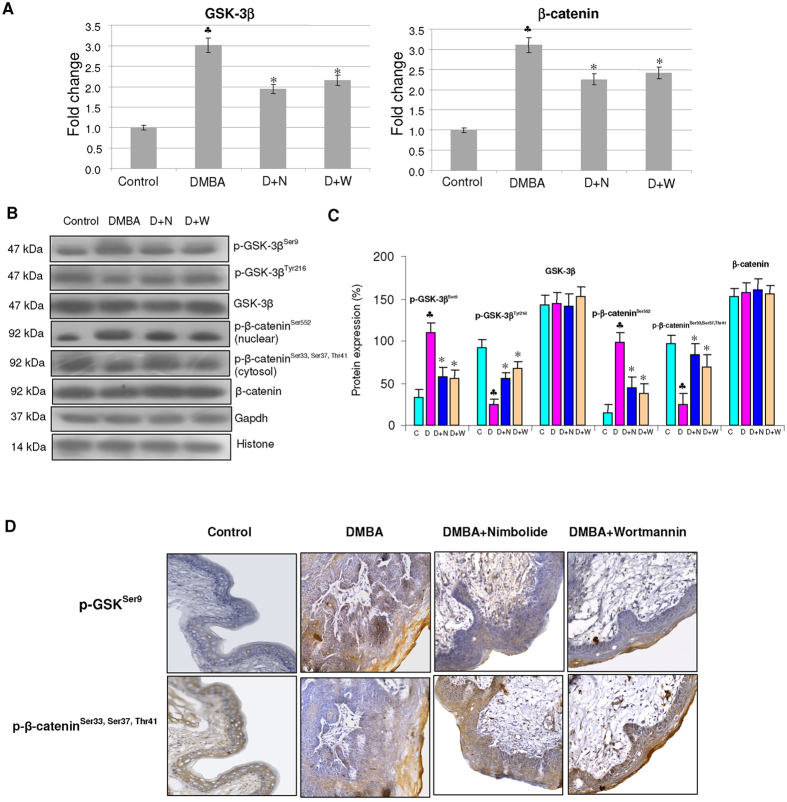
Effect of nimbolide on the mRNA and protein expression of GSK-3β and β-catenin in the hamster buccal pouch. (mean ± SD; n = 3). (**A**) Quantitative RT-PCR analysis of GSK-3β and β-catenin. The fold change in transcript expression for each gene was determined using the 2−ΔΔCt method. Data are the mean ± SD of three separate experiments. Statistical significance was determined by the Mann–Whitney test (p < 0.05). (**B**) Western blots showing expression levels of phosphorylated and unphosphorylated GSK-3β and β-catenin in control and experimental animals. Gapdh and histone H2B were used as loading control for cytosol and nuclear proteins respectively. (**C**) Background subtracted protein bands were quantified and normalized to Gapdh. Phosphorylated proteins are normalized by their unphosphorylated forms. Each bar represents the protein expression ± SD of three determinations. (**D**) Immunohistochemical analysis of p-GSK-3β^Ser9^ and p-β-catenin ^Ser33,Ser37,Thr41^. ^♣^ significantly different from control (p < 0.05). *significantly different from DMBA-treated group (p < 0.05) The groups are indicated as C (Control), D (Hamsters painted with DMBA for 12 weeks), D + N (Hamsters painted with DMBA for 12 weeks followed by nimbolide administration from 12 to 16 weeks), D + N (Hamsters painted with DMBA for 12 weeks followed by wortmannin administration from 12 to 16 weeks).

**Figure 5:**
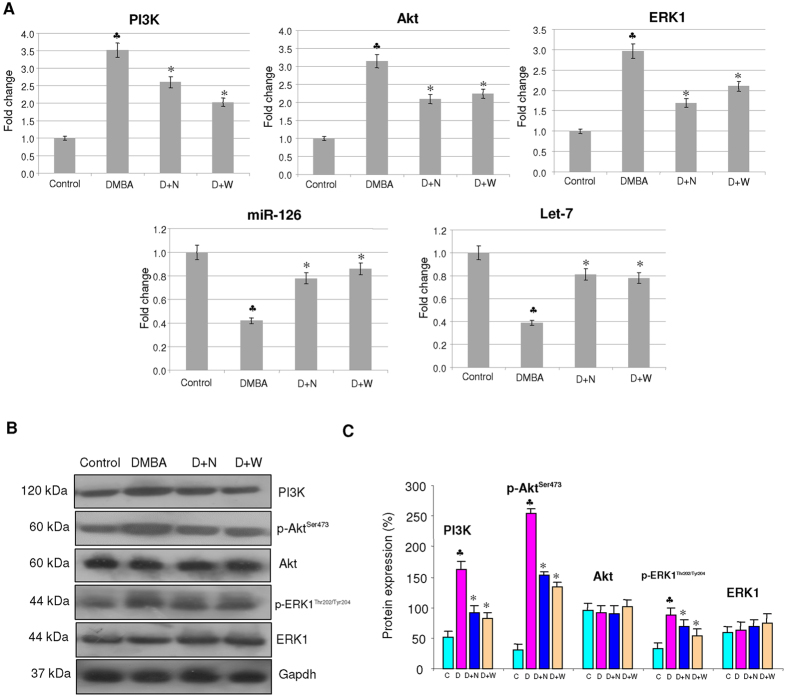
Effect of nimbolide on the mRNA and protein expression of PI3K, Akt, ERK, miR-126 and Let-7. (**A**) Quantitative RT-PCR analysis of PI3K, Akt, ERK, miR-126 and Let-7. Data are the mean ± SD of three separate experiments. Statistical significance was determined by the Mann–Whitney test (p < 0.05). (**B**) Immunoblot analysis of PI3K/Akt and ERK signalling molecules. Decreased expression of PI3K, p-Akt^Ser473^ and p-ERK1/2^Thr202/Tyr204^ observed in nimbolide treated animals compared to DMBA painted hamsters. Gapdh was used as loading control for cytosol. Phosphorylated proteins are normalized by their unphosphorylated forms. (**C**) Background subtracted protein bands quantified and normalized to Gapdh. Phosphorylated proteins are normalized by their unphosphorylated forms. Each bar represents the protein expression ± SD of three determinations. ^♣^significantly different from control (p < 0.05). *significantly different from DMBA-treated group (p < 0.05).

Administration of nimbolide inhibited nuclear translocation of β-catenin by activating GSK-3β. We observed a negative correlation between GSK-3β^Ser9^ and β-catenin that was confirmed by immunohistochemical analysis (Fig. 4). Nimbolide administration blocked activation of the upstream kinases PI3K, Akt and ERK. We also examined the effect of nimbolide on inducers of GSK-3β, miR-126 and Let-7. Our results demonstrate a significant increase in the expression of both miR-126 and Let-7 (Fig. 5).

### Nimbolide suppresses cell proliferation and induces apoptosis

**Figure 6:**
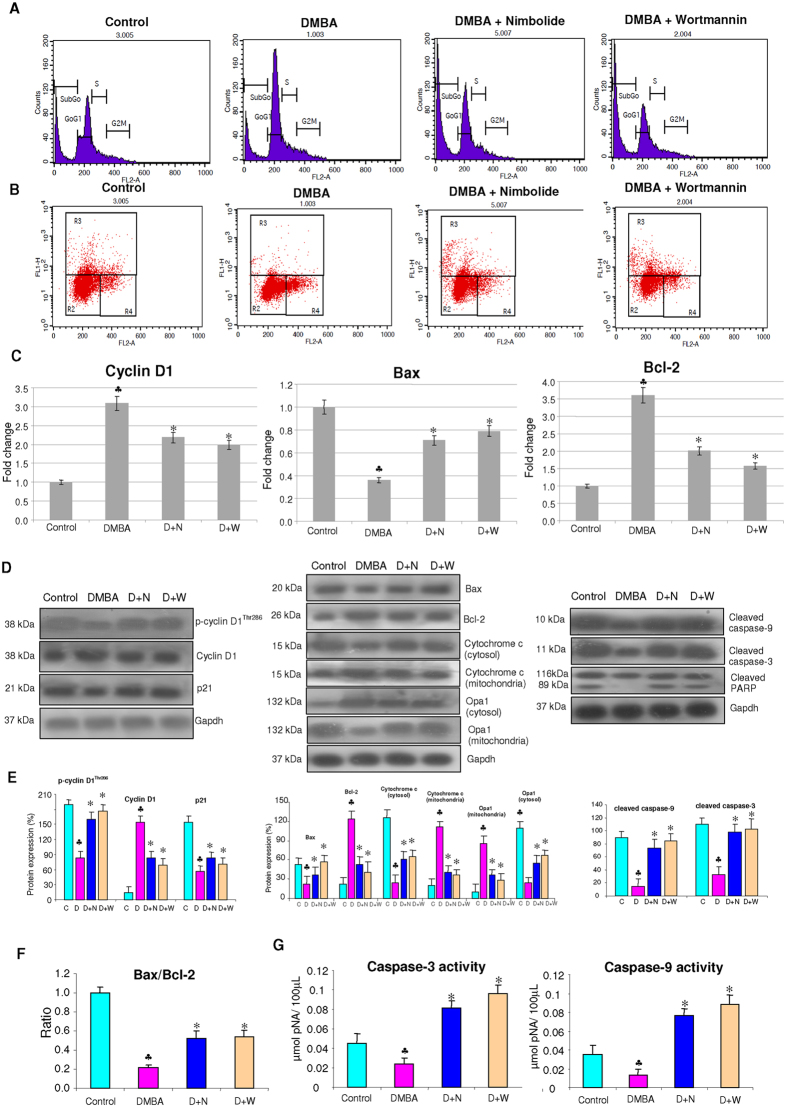
Effect of nimbolide on the mRNA and protein expression of markers of cell proliferation and apoptosis. (**A**) Flow cytometry analysis of the cell cycle in nimbolide and wortmannin treated animals. SubG0 cells represent apoptotic cells with lower DNA content. The data presented are representative of three independent experiments. (**B**) TUNEL assay by flow cytometry in hamster buccal pouch tissues. R3 region shows TUNEL positive cells. (**C**) Quantitative RT-PCR analysis of cyclin D1, Bax and Bcl-2. Data are the mean ± SD of three separate experiments. Statistical significance was determined by the Mann–Whitney test (p < 0.05). (**D**) Expression of proteins involved in cell cycle and apoptosis were analysed by western blotting. Gapdh was used as loading control. (**E**) Background subtracted protein bands quantified and normalized to Gapdh. Each bar represents the protein expression ± SD of three determinations. (**F**) Histogram representing Bax/Bcl-2 ratio in different groups. (**G**) The activities of caspase-3 and caspase-9 using colorimetric assay kits. Enzyme activities were significantly increased in nimbolide and wortmannin treated animals compared to DMBA painted hamsters. ^♣^ significantly different from control (p < 0.05). * significantly different from DMBA-treated group (p < 0.05).

We next analysed the impact of nimbolide administration on cell proliferation and apoptosis, the major downstream events influenced by GSK-3β/β-catenin (Fig. 6). Analysis of cell cycle progression by FACS revealed increased accumulation of cells in subG0/G1 phase with depletion of cells in S and G2/M phase indicating that nimbolide administration arrests the cell cycle in G1/S phase. To determine whether nimbolide-induced cell cycle arrest was caused by alterations in cell cycle regulatory proteins, we analysed the expression of cyclin D1 and p21. Since GSK-3β is known to phosphorylate and inactivate cyclin D1, we also assessed the expression levels of p-cyclin D1^Thr286^. Our results revealed downregulation of cyclin D1 with upregulation of the CDK inhibitor p21. Enhanced expression of p-cyclin D1^Thr286^ confirmed the presence of active GSK-3β in nimbolide treated hamsters.

Presence of subdiploid peak with increase in the proportion of cells with reduced DNA content indicated apoptosis induction by nimbolide. These results were further validated by TUNEL assay which showed 10.05% and 11.9% TUNEL positive cells in nimbolide and wortmannin treated groups respectively. In order to ascertain cell death by apoptosis, we analysed the expression of anti-apoptotic and pro-apoptotic proteins. Administration of nimbolide to DMBA painted animals showed upregulation of pro-apoptotic Bax with downregulation of anti-apoptotic Bcl-2. This was associated with increased expression of Opa-1, a dynamin-related mitochondrial protein involved in cristae remodelling and release of apoptogenic molecules. An increase in the expression of cytosolic cytochrome c relative to the mitochondrial fraction confirmed that nimbolide transduced apoptosis through the mitochondrial pathway. In addition, nimbolide increased the expression and activities of caspase-3 and −9 and induced cleavage of PARP.

### Nimbolide docks to key signalling kinases

**Figure 7:**
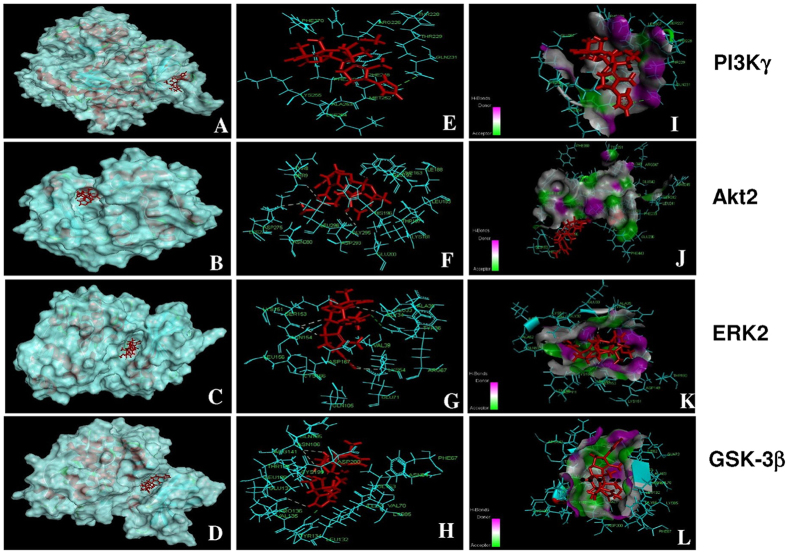
Docking analysis of nimbolide with PI3Kγ, Akt, ERK and GSK-3β. (**A–D**) represent computational modelling of nimbolide (red sticks) binding to PI3Kγ RAS binding domain and kinase domains of Akt2, ERK2, GSK-3β. (**E–H**) Hydrogen bonds are indicated as green dotted lines between nimbolide and PI3Kγ, Akt2, ERK2, GSK-3β. (**I–L**) Surface representation of PI3Kγ, Akt2, ERK2, GSK-3β complexed to nimbolide (red sticks) illustrating the locations of hydrogen bond donor amino acids (pink) and acceptor amino acids (green).

Molecular docking analysis revealed hydrogen bonds between nimbolide and Tyr-134 and Gln-185 residues on the kinase domain of GSK-3β with a docking score of 32.73 confirming its modulatory influence on Wnt signalling. The results of the present study also reveal interactions of nimbolide with PI3K, Akt and ERK. Nimbolide formed hydrogen bonds with Gln 231 in the RAS binding domain (RBD) of PI3Kγ, Glu 193, Asp 275 and Gly 295 residues in the kinase domain of Akt 2, and Gly 34, Lys 54, Arg 67, Glu 71 and Ser 153 in the kinase domain of ERK2 (Fig. 7 and Supplementary Table S3).

## Discussion

Dysregulation of GSK-3β has been extensively documented in a wide range of malignancies including oral cancer^11^,^25^,^26^,^27^,^28^. Here, we report for the first time, inactivation of GSK-3β during the stepwise evolution of HBP carcinomas based on increased levels of p-GSK-3β^Ser9^ with concomitant decrease in p-GSK-3β^Tyr216^. Furthermore, we found a progressive shift of p-GSK-3β^Ser9^ to the nucleus associated with increase in p-β-catenin^Ser552^ during progression from hyperplasia through dysplasia to carcinoma confirming the inactivation of GSK-3β. Phosphorylation of β-catenin at Ser^552^ by Akt has been reported to induce its transcriptional activity^29^. Interestingly, nimbolide decreased p-GSK-3β^Ser9^, the inactive form of GSK-3β and induced cytoplasmic accumulation of active GSK-3β. In addition, nimbolide also increased the expression of miR-126 and let-7 that are known to stimulate GSK-3β activity thereby facilitating degradation of β-catenin^30^. Molecular docking studies confirmed interaction of nimbolide with Tyr-134 and Gln-185 residues in the kinase domain of GSK-3β. This may account for increased p-β-catenin^Ser33,37,Thr41^ in hamsters administered nimbolide that could prevent nuclear translocation of β-catenin and consequent transactivation of genes involved in cell proliferation and apoptosis evasion.

Cyclin D1, a proto-oncogene and a master transcriptional regulator that functions at the crossroads of several cellular processes including cell proliferation and apoptosis is a key substrate for GSK-3β^31^. GSK-3β phosphorylates cyclin D1 at Thr^286^, a reaction that promotes the redistribution of cyclin D1 from the nucleus to the cytoplasm and subsequent degradation^32^. Recently, we demonstrated a positive correlation between GSK-3β inactivation and enhanced expression of cyclin D1 in OSCC^28^. Dysregulated phosphorylation of GSK-3β observed in HBP carcinomas in the present study can prevent Thr^286^ phosphorylation leading to nuclear accumulation of cyclin D1 and cell cycle progression. Notably, administration of nimbolide reduced the expression, activity, nuclear localization and phosphorylation of cyclin D1 whilst simultaneously upregulating its negative regulator p21. Downregulation of cyclin D1 was accompanied by accumulation of cells in subG0/G1 indicating that nimbolide blocks cell cycle progression and directs cells for apoptosis.

The results of the present study provide evidence that nimbolide induces the mitochondrial pathway of apoptosis as reflected by enhanced Bax/Bcl-2 ratio, efflux of cytochrome c from the mitochondria into the cytosol, increased caspase-3 activity and cleavage of PARP, an endogenous substrate of caspase-3. In addition, DNA fragmentation, a characteristic hallmark of apoptosis, as revealed by TUNEL assay, further confirms apoptosis induction by nimbolide. These results are consistent with previous reports by us and others that demonstrated cell cycle arrest and modulation of apoptosis regulating proteins by nimbolide both *in vitro* and *in vivo*^17^,^19^,^20^,^21^,^22^,^23^,^33^. Blocking GSK-3β phosphorylation at Ser^9^ has been reported to attenuate cell proliferation while concomitantly stimulating caspase-3 mediated apoptosis in glioblastoma stem-like cells^34^. Likewise, nimbolide by targeting GSK-3β and β-catenin appears to influence the opposing pathways of cell proliferation and apoptosis that may in turn be regulated by upstream signalling kinases.

The serine threonine kinases Akt and the MAPKs are among the most important kinases that inactivate GSK-3β by site-specific phosphorylation at Ser^9^,^11^,^12^. Aberrant expression of these kinases during the sequential progression of HBP carcinomas seen in the present study is in line with constitutive activation of these pathways in a wide range of malignant tumours including oral carcinomas^35^,^36^,^37^,^38^. Akt, the main downstream target of PI3K consists of three conserved domains including N-terminal pleckstrin homology domain, central kinase catalytic domain, and C-terminal extension domain^12^. The interaction of nimbolide with the kinase domain of Akt may impede the translocation of Akt from the cytoplasm to the plasma membrane and consequent PDK-1 mediated phosphorylation at Ser^473^ vital for its activation. In a recent study, Karkare *et al.*, demonstrated that nimbolide blocks growth factor-induced phosphorylation of Akt in glioblastoma multiforme cells and in tumour xenografts supporting reduced expression of p-Akt observed in the present study^39^.

PI3Kγ that plays a vital role in the regulation of cell survival and proliferation via transcription factors and the MAPK pathways consists of five domains including adapter binding domain (ABD), Ras-binding domain (RBD), C2 domain, helical domain and catalytic domain. Several direct inhibitors of PI3Kγ such as wortmannin, curcumin, quercetin, myricetin, staurosporine and GSK2126458 target the kinase domain^40^,^41^. Here we show that nimbolide docks to Gln 231 on the RAS binding domain of PI3Kγ that could prevent binding of RAS essential for PI3Kγ activation and further downstream signalling. Recent reports indicate that nimbolide acts as a potent anticancer agent by inducing apoptosis and inhibiting cell proliferation via the PI3K/Akt pathway in PC-3 cells lending credence to similar observations in the HBP model^42^.

**Figure 8:**
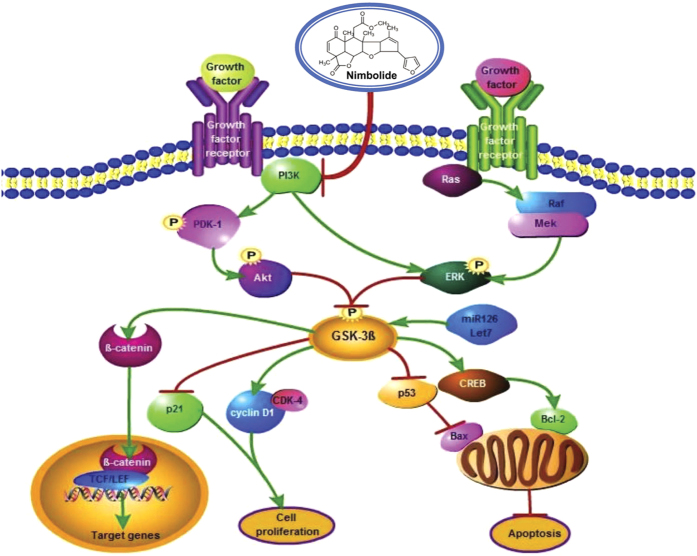
Schematic representation of the mechanism of action of nimbolide in DMBA induced oral carcinomas. Dietary administration of nimbolide inhibits DMBA induced oral cancer by targeting PI3K. Nimbolide mediated inhibition of PI3K abrogates ERK expression and increases the expression of GSK-3β and Let-7, miR126 eventually culminating in inhibition of cell proliferation and evasion of apoptosis.

The MAPKs, ERK1 and ERK2 participate in the regulation of a variety of cellular processes including cell adhesion, cell cycle progression, cell migration, cell survival, differentiation, metabolism, proliferation, and transcription^43^,^44^. Babykutty *et al.* demonstrated that nimbolide induces caspase-mediated apoptosis by inhibiting ERK1/2 and activating p38 and JNK1/2^45^. Molecular docking analysis revealed binding of nimbolide to Gly 34, Lys 54, Arg 67, Glu 71 and Ser 153 of ERK2 in the kinase domain accounting for the kinase inhibitory effects of nimbolide. Figure 8 summarises the mechanism by which nimbolide exerts its chemotherapeutic potential in the HBP model.

Taken together, the results of the present study demonstrate PI3K/Akt and MAPK driven dysregulation of GSK-3β with consequent aberrant cell proliferation and apoptosis during oncogenic progression in the HBP model. Our study also provides compelling evidence that the modulatory effects of nimbolide on GSK-3β are secondary to blockade of upstream kinase signalling, primarily the PI3K/Akt pathway. Thus the neem limonoid nimbolide, a potent PI3K/Akt inhibitor may be a useful addition to the armamentarium of natural compounds for oral cancer treatment.

## Materials and Methods

### Chemicals

Acrylamide, bovine serum albumin (BSA), bromophenol blue, DMBA, ethidium bromide, 2-mercaptoethanol, sodium dodecyl sulphate (SDS), N,N,N’,N’-tetramethylene diamine (TEMED) and trizol were purchased from Sigma Chemical Company, St. Louis, MO, USA. Power SYBR^®^ Green PCR master mix was obtained from Applied Biosystems, California, USA. Antibodies for cyclin D1, CDK4, p21, p53, GSK-3β, p-GSK-3β^Tyr216^, Akt, PI3K, cleaved caspase-3, cleaved caspase-9, cytochrome c, Opa-1 and Gapdh were procured from Santa Cruz Biotechnology, USA. Antibodies for Bcl-2, Bax, p-Akt^Ser473^, β-catenin, p-β-catenin^Ser33,Ser37,Thr41^, p-β-catenin^Ser552^, CREB, p-GSK-3β^Ser9^, ERK1, p-ERK1^Thr202/Tyr204^, p-cyclin D1^Thr286^, cleaved PARP and histone as well as ELISA kits were from Cell Signaling Technology, USA. Oligonucleotide primers and primers for mature microRNA were procured from Sigma Genosys, San Ramon, USA (The primer details are provided in Supplementary Table S4). Nimbolide was obtained from M/s Asthagiri Herbal Research Foundation, Chennai, India. Wortmannin was obtained from Selleckchem, USA. All other reagents used were of analytical grade.

### Animals and diet

The experiments were carried out using male Syrian hamsters of 8–10 weeks old weighing between 100 and 110 g, procured from the National Centre for Laboratory Animal Sciences (NCLAS), NIN, India. The animals were accommodated six to a polypropylene cage and provided with standard pellet diet (Sai Enterprisei, Chennai, India) and water ad libitum. The animals were maintained in a controlled environment under standard conditions of temperature and humidity with an alternating 12 h light/dark cycle in accordance with the guidelines of the Indian Council of Medical Research. All experimental procedure was approved by the Institutional Animal Ethics Committee, Annamalai University, and conducted according to the guidelines by the Committee for the Purpose of Control and Supervision on Experiments on Animals (CPCSEA).

### Treatment schedule

The animals were randomized into experimental and control groups and divided into 5 groups of 6 animals each. Animals in group 1 received basal diet alone and served as control. The right buccal pouches of hamsters in the experimental groups 2–5 were painted with a 0.5% solution of DMBA in liquid paraffin, three times per week for 4, 8, 12, and 16 weeks, respectively^25^,^46^. At the end of 0, 4, 8, 12, and 16 weeks of DMBA application, animals in the respective groups were sacrificed by cervical dislocation after an overnight fast. Before an animal was killed, the right pouch was grossly inspected to evaluate premalignant lesions and tumour development. A portion of the buccal pouch tissue was immediately frozen in liquid nitrogen for subsequent RNA extraction, while another portion was processed using lysis buffer for western blot analysis.

A second set of experiments was carried out to assess the chemotherapeutic effect of nimbolide. The animals were divided into 4 groups of 6 animals each. Hamsters in group 1 served as untreated control. The right buccal pouches of hamsters in group 2 were painted with 0.5% DMBA for 12 weeks followed by a basal diet up to the 16th week. Animals in groups 3 and 4 painted with DMBA as in group 2, received in addition, intragastric administration of nimbolide and wortmannin daily at a concentration of 100 and 500 μg/kg bw respectively from the 12th to the 16th week^17^,^47^. The experiment was terminated after 16 weeks and all animals were sacrificed by cervical dislocation after an overnight fast.

### Quantitative real-time RT-PCR (qRT-PCR) for mRNA and microRNA expression analysis

Total RNA was isolated from the buccal pouch tissues by the method of Chomczynski and Sacchi^48^. About 5 μg of the isolated RNA was reverse-transcribed to cDNA in a reaction mixture containing 4 μl of 5× reaction buffer, 2 μl of dNTPs mixture (10 mM), 20 units of RNase inhibitor, 200 units of avian-myeloblastosis virus (AMV) reverse transcriptase and 0.5 μg of oligo(dT) primer (Promega, WI, USA) in a total volume of 20 μl. The reaction mixture was incubated at 42 ^o^C for 60 min and the reaction terminated by heating at 70 ^o^C for 10 min. The cDNA was stored at −80 ^o^C until further use.

MicroRNA was isolated from tissues using miRNeasy minikit method (Qiagen) according to the manufacturer’s protocol and quantified using a Biophotometer. cDNA was synthesised using NCode^TM^ VILO^TM^ miRNA cDNA Synthesis Kit (Invitrogen).

Quantitative RT-PCR was performed in a StepOnePlus thermocycler (Applied Biosystems) using Power SYBR Green. To the 1× PCR master mix, 2.5 μl of the cDNA was added in a total volume of 20 μl. The PCR conditions were as follows: 95 ^o^C for 5 min, 40 cycles of 30 s at 95 ^o^C, 30 s at 52–60 ^o^C (based on the target) and 60 s at 72 ^o^C. Relative quantitative fold change was calculated using the comparative Ct method.

### Protein extraction and Western blotting

For extracting the total protein, approximately 50 mg of each tissue sample was subjected to lysis in a sample buffer containing 62.5 mM Tris (pH 6.8), 2% SDS, 5% 2-mercaptoethanol, 10% glycerol, and bromophenol blue. Nuclear and cytoplasmic extracts were prepared as described by Legrand-Poels *et al.*^49^. Tissue samples were homogenized with 1 mL of a buffer containing 10 mM 4-(2-hydroxyethyl) piperazine-1-ethanesulfonic acid (HEPES), 10 mM KCl, 0.1 mM EDTA, 0.1 mM ethylene glycol tetraacetic acid (EGTA), 1 mM dithiothreitol (DTT), 0.5 mM phenylmethylsulfonyl fluoride (PMSF), and 10 μL protease inhibitor cocktail. The lysate was then centrifuged at 8,000 × g for 2 min at 4 °C, and the supernatant containing the cytoplasmic fraction was removed, aliquoted, and frozen at −80 °C. The nuclear pellet was reconstituted in 1 mL of buffer containing 20 mM HEPES (pH 7.9), 0.4 M sodium chloride, 1 mM EDTA, 1 mM EGTA, 1 mM DTT, 1 mM PMSF, and 10 μL protease inhibitor cocktail, followed by vigorous vortexing for 20 min at 4 °C. The nuclear lysate was then centrifuged at 14,000 × g for 5 min, and nuclear extracts were aliquoted and stored at −80 °C. For isolating the mitochondrial fraction, the tissue lysate was centrifuged at 1,000 × g for 5 min to remove the nucleus and unbroken cells. The supernatant was centrifuged at 23,100 × g for 30 min at 4 °C, and the resulting pellet containing the mitochondria was suspended in lysis buffer (150 mM NaCl, 0.5% Triton X-100, 50 mM Tris, 20 mM EGTA, 1 mM DTT, 1 mM sodium orthovanadate, and protease inhibitor cocktail)^50^.

The protein samples were electrophoresed on SDS-PAGE, the resolved proteins transferred to PVDF membrane and probed with corresponding primary and secondary antibodies as described previously^15^. After extensive washes with high and low salt buffers, the immunoreactive proteins were visualized using enhanced chemiluminescence (ECL) detection reagents (Sigma). Densitometry was performed on IISP flatbed scanner and quantitated with Total Lab 1.11 software. The phospho forms of proteins were normalized with their respective unphosphorylated counterparts.

### Assay of caspase-3 and caspase-9 activities

The activities of caspases were assayed using caspase-3 (Sigma, St. Louis MO, USA) and caspase-9 (Calbiochem, USA) colorimetric assay kits according to the manufacturer’s instructions. Cytosolic extracts were prepared by homogenizing tissues in lysis buffer containing 50 mM HEPES (pH 7.4), 5 mM 3-[(3-cholamidopropyl) dimethylammonio]-1-propanesulfonate (CHAPS) and 5 mM DTT. The supernatant was collected as an enzyme source. The assays are based on the hydrolysis of the peptide substrate acetyl-Asp-Glu-Val-Asp-nitroanilide (Ac-DEVD-pNA) by caspase-3, and Leu-Glu-His-Asp-nitroanilide (LEHD-pNA) by caspase-9 and subsequent release of the chromophore p-nitroaniline (pNA). The concentration of pNA released from the substrate was calculated from the absorbance values at 405 nm.

### Cell cycle analysis and TUNEL assay

Cells were isolated from tissue samples and filtered using a cell strainer for monosuspension. The cells were then gently fixed with 70% ice cold ethanol at−20 °C for 1 h, suspended in PBS containing 0.5 μg/ml RNase, and incubated at 37 °C for 30 min. Following this, cells were stained with 50 μg/ml propidium iodide for 30 min, and the DNA content was analyzed on a flow cytometer (FACS Caliber Flow Cytometer, Becton Dickinson). TUNEL assay was performed using APO-BrdU TUNEL assay kit (Invitrogen) as per the manufacturer’s instructions. At the end of the assay, cells were subjected to flow cytometry to determine percent of apoptotic cells. A minimum of 20,000 cells were collected for each measurement.

### Immunohistochemistry

Paraffin embedded tissue sections were deparaffinised by heating at 60 ^o^C for 10 min, followed by three washes with xylene. After gradual hydration through graded alcohol, the slides were incubated in citrate buffer (pH 6.0) for two cycles for 5 min in a microwave oven for antigen retrieval. The sections were allowed to cool for 20 min and then rinsed with Tris-buffered saline (TBS). The sections were treated for 15 min with 3% H_2_O_2_ in distilled water to inhibit endogenous peroxidase activity. Non-specific antibody binding was reduced by incubating the sections with Universal Power Block (BioGenex, San Ramon, CA, USA) for 10 min. The sections were then incubated with primary antibodies at room temperature for 3 h. The slides were washed with TBS and then incubated with biotin-labeled secondary antibody followed by streptavidin-biotin-peroxidase (Dako, Carprinteria, CA, USA) for 30 min each at room temperature. The immunoprecipitate was visualized by treating with 3, 3′-diaminobenzidine and counterstaining with hematoxylin. The tissues were then photographed using an Inverted Fluorescent Microscope (Leica Microsystem Vertrieb GmbH, Wetzler, Germany) attached with digital camera DFC295.

### Molecular docking

Molecular docking was performed by using Discovery Studio 2.7 package from Biosystems Technologies, San Diego, USA on O2 (R12000) workstation. Nimbolide was retrieved from PubChem (www.ncbi.nlm.nih.gov/pccompound) (CID 100017). X-ray crystal structures of oncogenic signalling kinases PI3Kγ (1E8Y), Akt2 (2JDO), ERK2 (1TVO), and GSK-3β (4ACC) were retrieved from Protein Data Bank (PDB) (www.rcsb.org). Nimbolide ligand structure was minimized by charmM force field, and the stable energy conformation was taken for docking studies. The retrieved proteins were processed by removing heteroatoms, metal ions and water molecules, followed by addition of H-bonds to satisfy valencies and minimized by charmM force field. Docking analysis was carried out using Ligandfit module of Accelrys Discovery Studio (ADS). The ligand nimbolide was docked independently with the receptor proteins. The targeted protein was built as a receptor molecule to predict the active binding site and compute interaction parameters with the ligand. After docking, poses were viewed and analysed by DS Visualizer 3.5.

### Statistical analysis

Statistical analysis was carried out using a nonparametric Mann-Whitney test (StatsDirect, United Kingdom). A probability value of less than 0.05 was considered significant.

## Additional Information

**How to cite this article**: Sophia, J. *et al.* Nimbolide, a neem limonoid inhibits Phosphatidyl Inositol-3 Kinase to activate Glycogen Synthase Kinase-3β in a hamster model of oral oncogenesis. *Sci. Rep.* **6**, 22192; doi: 10.1038/srep22192 (2016).

## Supplementary Material

Supplementary figure s1-s4
