# Supplementary material for: Nimbolide, a neem limonoid inhibits Phosphatidyl Inositol-3 Kinase to activate Glycogen Synthase Kinase-3β in a hamster model of oral oncogenesis
Source: Sci Rep. 2016 Feb 23;6:22192. doi: 10.1038/srep22192 (PMC4763291; doi:10.1038/srep22192)
Supplement: Supplementary figure s1-s4 [file srep22192-s1.doc]

**Nimbolide, a neem limonoid inhibits Phosphatidyl Inositol-3 Kinase to activate Glycogen Synthase Kinase-3β in a hamster model of oral oncogenesis**

Josephraj Sophia ^1^, Kranthi Kiran Kishore T^1^, Jaganathan Kowshik^1^, Rajakishore Mishra^2^, Siddavaram Nagini^1^*****

**Supplementary Table S1.** **Tumour incidence and histopathological changes during sequential progression of OSCC (mean ± SD; n=6)**

| **Group** | **Treatment (Weeks)** | **Tumour multiplicity^a^** | **Tumour burden^b^ (mm^3^)** | **Hyperplasia** | **Dysplasia** | **SCC Incidence (%)** |
| --- | --- | --- | --- | --- | --- | --- |
| 1. | 0 week | - |  | - | - | - |
| 2. | DMBA - 4 weeks | - |  | ++ | - | - |
| 3. | DMBA - 8 weeks | - |  | +++ | +++ | - |
| 4. | DMBA - 12 weeks | 2.16 ± 0.16^^ | 42.66 ± 6.78^^ | +++ | +++ | 100 |
| 5. | DMBA - 16 weeks | 3.16 ± 0.68^^ | 91.83 ± 6.18^^ | +++ | +++ | 100 |

+ mild, ++ moderate, +++ severe, − no change, SCC squamous cell carcinoma

^a^Tumor multiplicity (number of tumors per hamster).

^b^Mean tumour burden was calculated by multiplying the mean tumour volume (4/3πr^3^) with the mean number of tumours (r=1/2 tumour diameter in mm)

^^Significantly different from control (p<0.05) by Mann–Whitney test

| **Group** | **Treatment** | **Body weight gained (g)** | **Tumour burden^a^ (mm^3^)** | **Tumour growth delay^b^ (%)** | **Hyperplasia** | **Dysplasia** |
| --- | --- | --- | --- | --- | --- | --- |
| 1. | Control | 48.8 ± 2.8 | - | - | - | - |
| 2. | DMBA | 21.7 ± 1.9^^ | 41.86 ± 5.67^^ | - | +++ | +++ |
| 3. | DMBA + Nimbolide (Post treatment) | 36.5 ± 1.6^*^ | 16.23 ± 8.31^*^ | 52.4 | ++ | ++ |
| 4. | DMBA + Wortmannin (Post treatment) | 39.2 ± 2.7^*^ | 14.88 ± 9.58^*^ | 46.8 | ++ | ++ |

**Supplementary Table S2.** **Effect on nimbolide and wortmannin on tumour incidence and histopathological changes (mean ± SD; n=6).**

+ mild, ++ moderate, +++ severe, − no change, SCC squamous cell carcinoma

^a^Mean tumour burden was calculated by multiplying the mean tumour volume (4/3πr^3^) with the mean number of tumours (r=1/2 tumour diameter in mm)

^b^Tumor growth delay was calculated by dividing differences in tumor volume (12th and 16th week) between DMBA treated group and chemotherapy group multiplied by 100.

^^Significantly different from control (p<0.05)

^*^Significantly different from DMBA-treated group (p<0.05).

**Supplementary Table S3. Docking score of nimbolide with PI3Kγ, Akt, ERK and GSK-3β**

| **S. No.** | **PDB ID** | **Nimbolide** | | **Bond Distance (**A◦) |
| --- | --- | --- | --- | --- |
|  |  | **Docking Score** | **Hydrogen bond** |  |
| 1 | 1E8Y/  PI3Kγ | 35.11 | A:GLN231:HE22 - 100017:O7 | 2.71572 |
| 2 | 2JDO/  Akt2 | 38.66 | A:GLY295:HA2 - 100017:O3  100017:H38 - A:GLU193:OE1  100017:H64 - A:ASP275:OD2 | 2.55459  3.02435  2.63603 |
| 3 | 1TVO/  ERK2 | 29.74 | A:ARG67:HH22 - 100017:O6  A:GLY34:HA1 - 100017:O4  A:LYS54:HE2 - 100017:O7  100017:H37 - A:SER153:O  100017:H39 - A:SER153:O  100017:H60 - A:GLU71:OE2 | 3.04542  2.47739  2.70448  2.68682  2.3804  2.34738 |
| 4 | 4ACC/  GSK-3β | 32.73 | A:TYR134:HH - 100017:O7  100017:H38 - A: GLN185:O | 2.85685  2.86112 |

**Supplementary Table S4. Primer sequences**

| **S.No** | **Gene product** | **Primers** | **Oligonucleotide sequence (5’-3’)** |
| --- | --- | --- | --- |
| 1. | Bax | Sense  Antisense | ACCAAGCTGAGCGAGTGTC  ACAAAGATGGTCACGGTCTGCC |
| 2. | Bcl-2 | Sense  Antisense | TGCACCTGACGCCCTTCAC  AGACAGCCAGGAGAAATCAAACAG |
| 3. | β-catenin | Sense  Antisense | CTTGCTCAGGGACAAGGAAGC  CTGGCCATATCCACCAGAGT |
| 4. | Cyclin D1 | Sense  Antisense | CGGAGGACAACAAACAGATC  GGGTGTGCAAGCCAGGTCCA |
| 5. | ERK1 | Sense  Antisense | GATTGCTGACCCTGAGCAC  GGGGGCCTCTGGTGCC |
| 6. | GSK-3β | Sense  Antisense | AGCCTATATCCATTCCTTGG  CCTCGGACCAGCTGCTTT |
| 7. | Let-7 | Sense  Antisense | CUAUACAACCUACUGCCUUCC  GGTGCATTGATTCCCGAGT |
| 8. | miR-126 | Sense  Antisense | GCAUUAUUACUCACGGUACGA  GGTGCATTGATTCCCGAGT |
| 9. | p21 | Sense  Antisense | CTCAGCGGAGGCGCCATG  GGGCGGATTAGGGCTTCC |
| 10. | PI3K | Sense  Antisense | TTAAACGCGAAGGCAACGA  CAGTCTCCTCCTGCTGTCGAT |


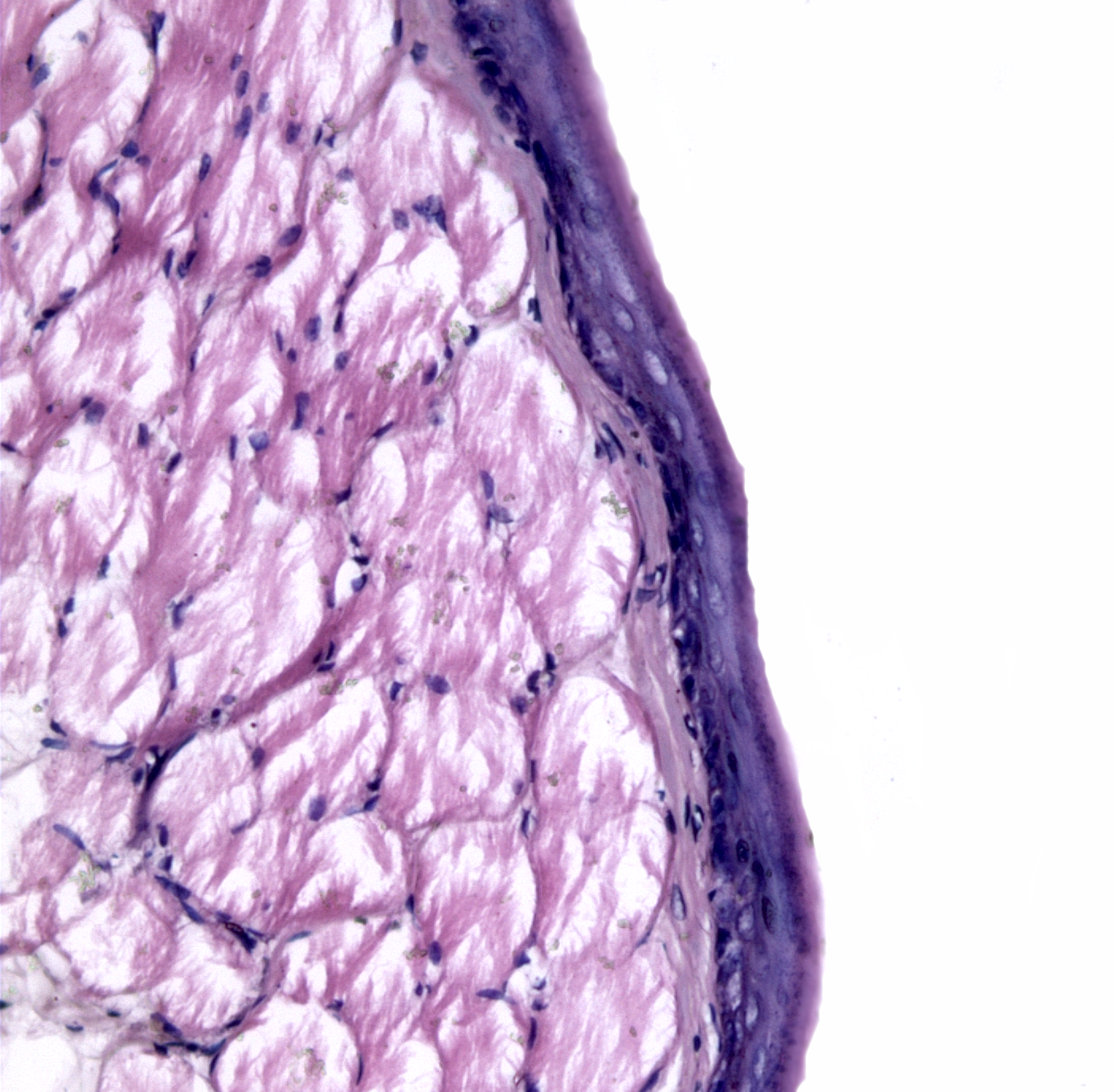


**A**


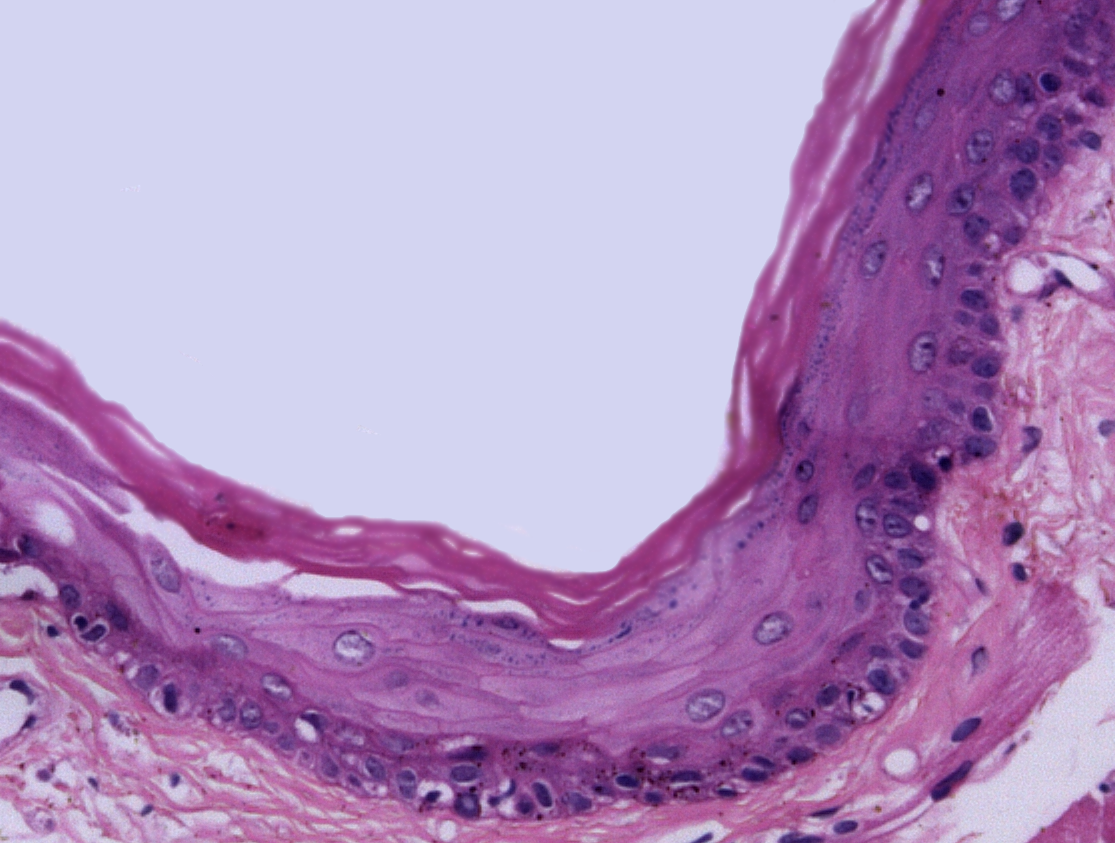


**B**


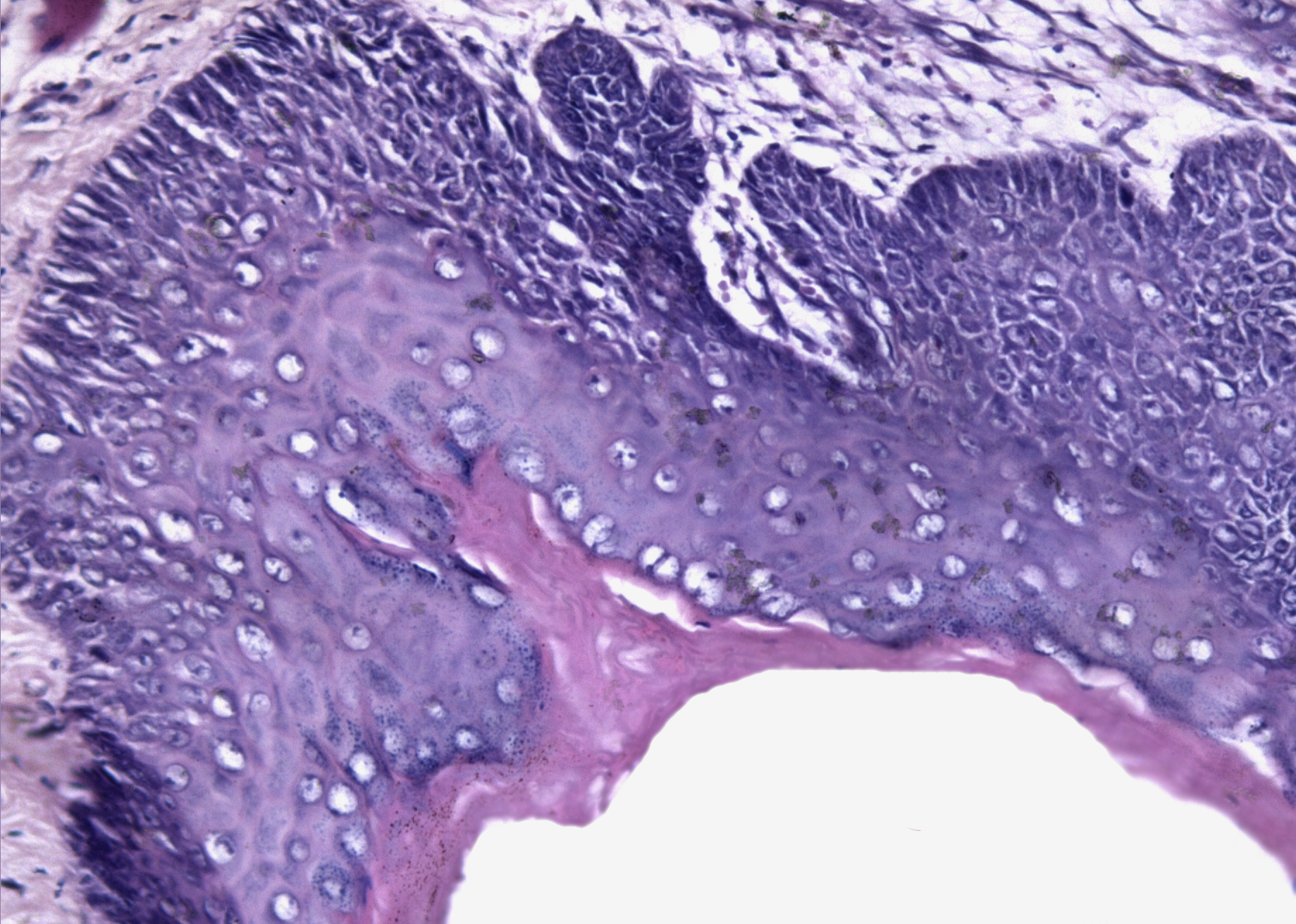


**D**


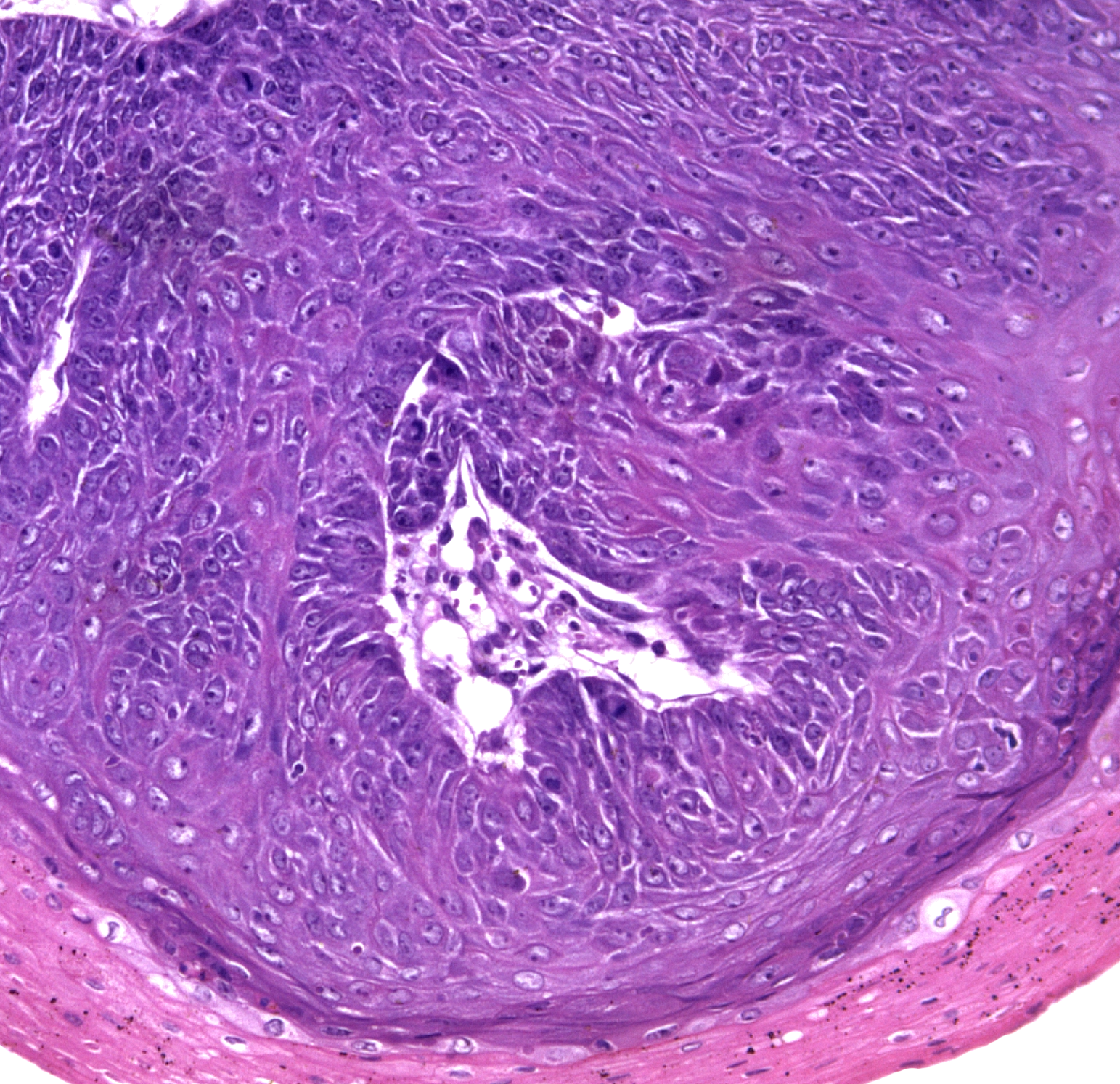


**E**


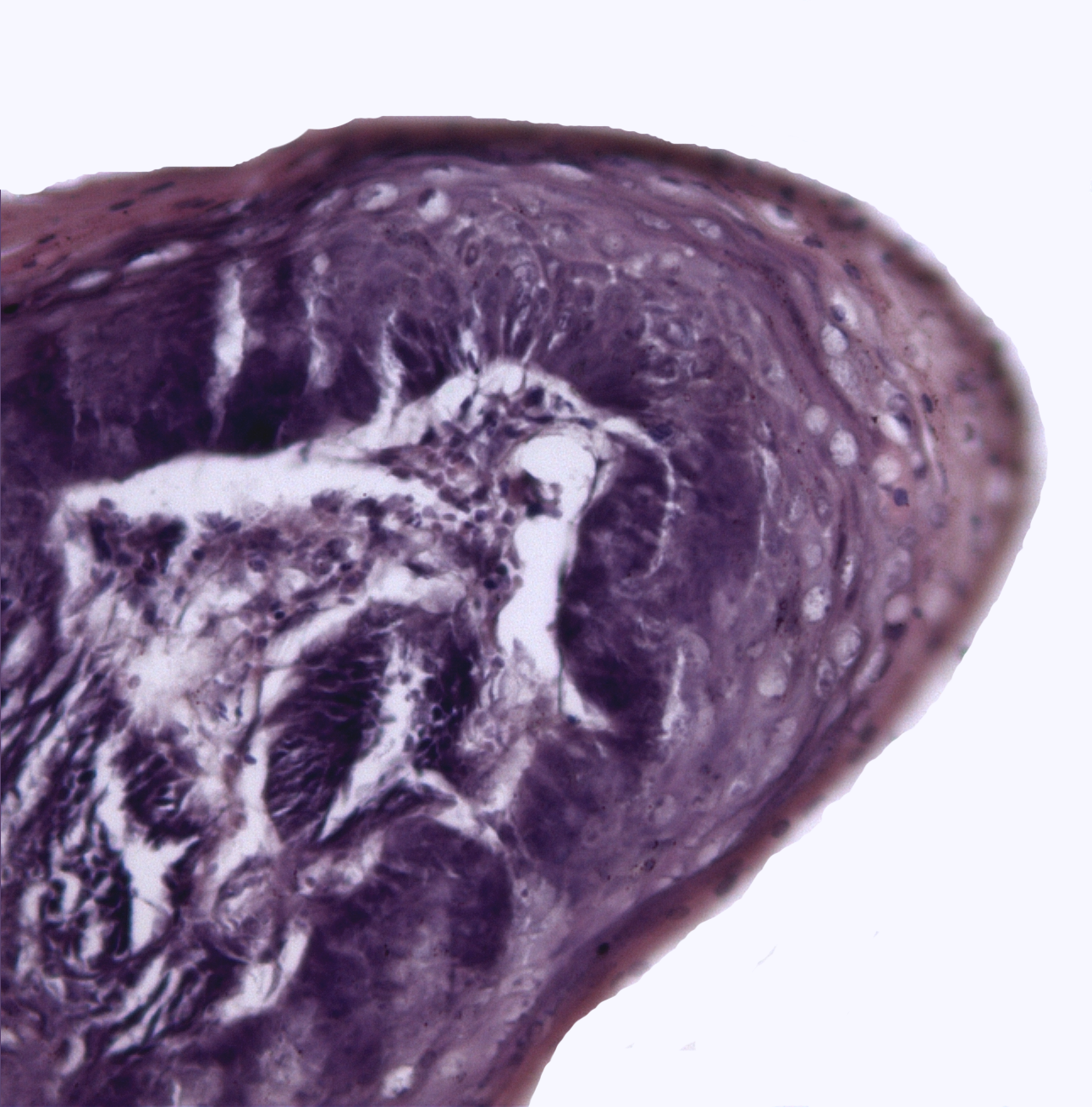


**C**

**i**

**ii**


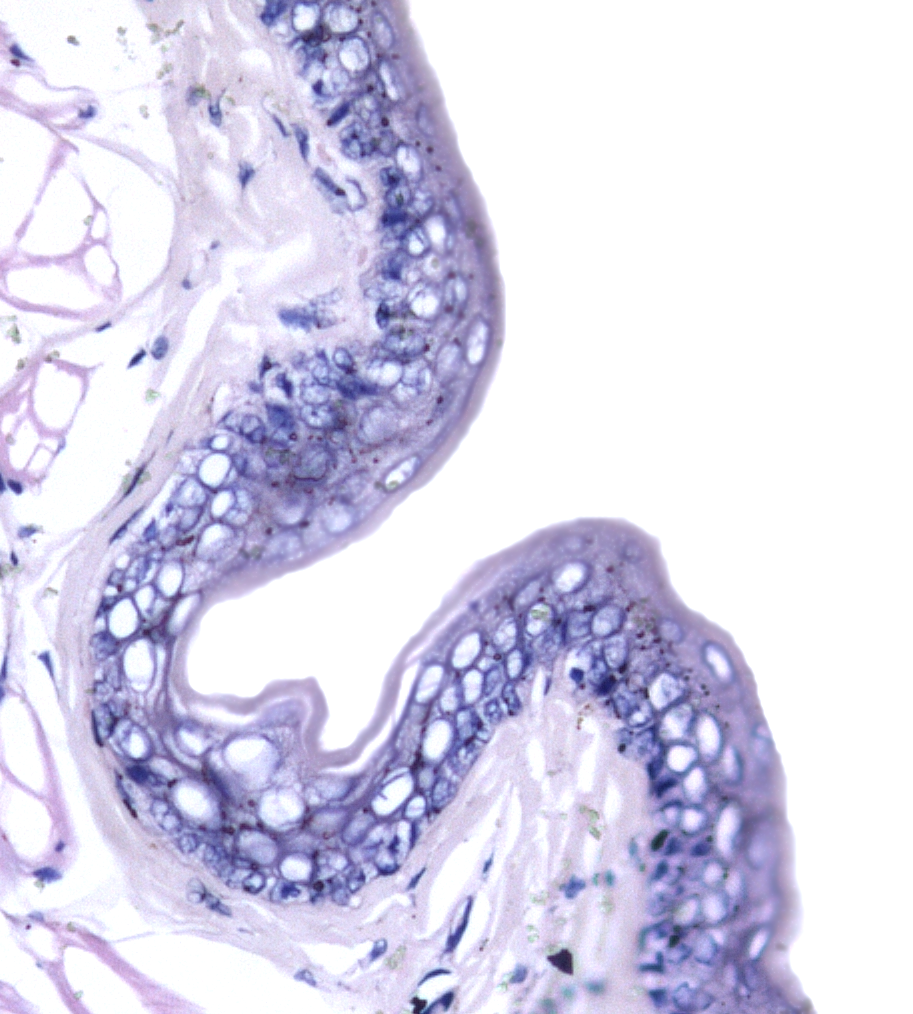


**A**


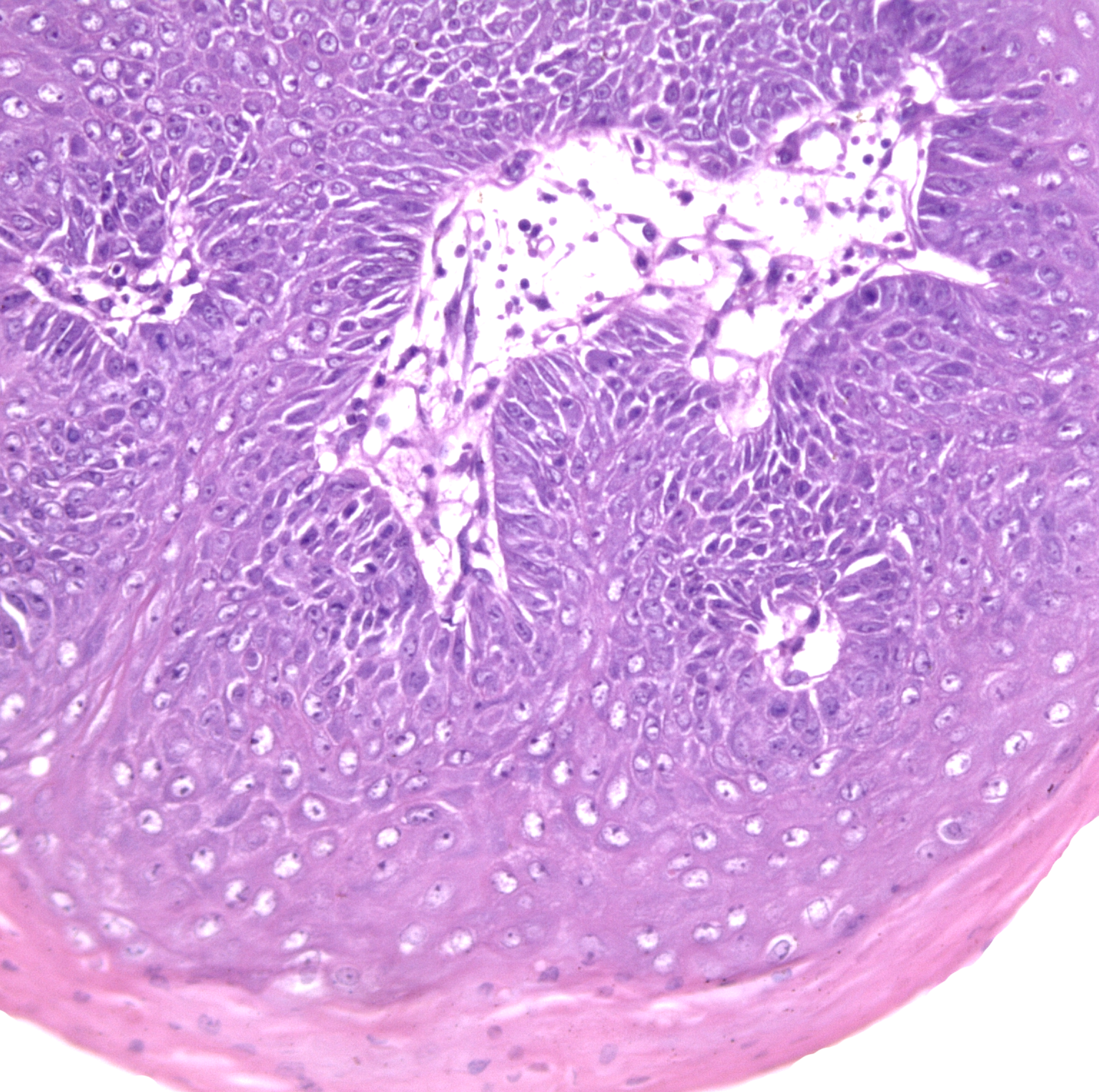


**B**


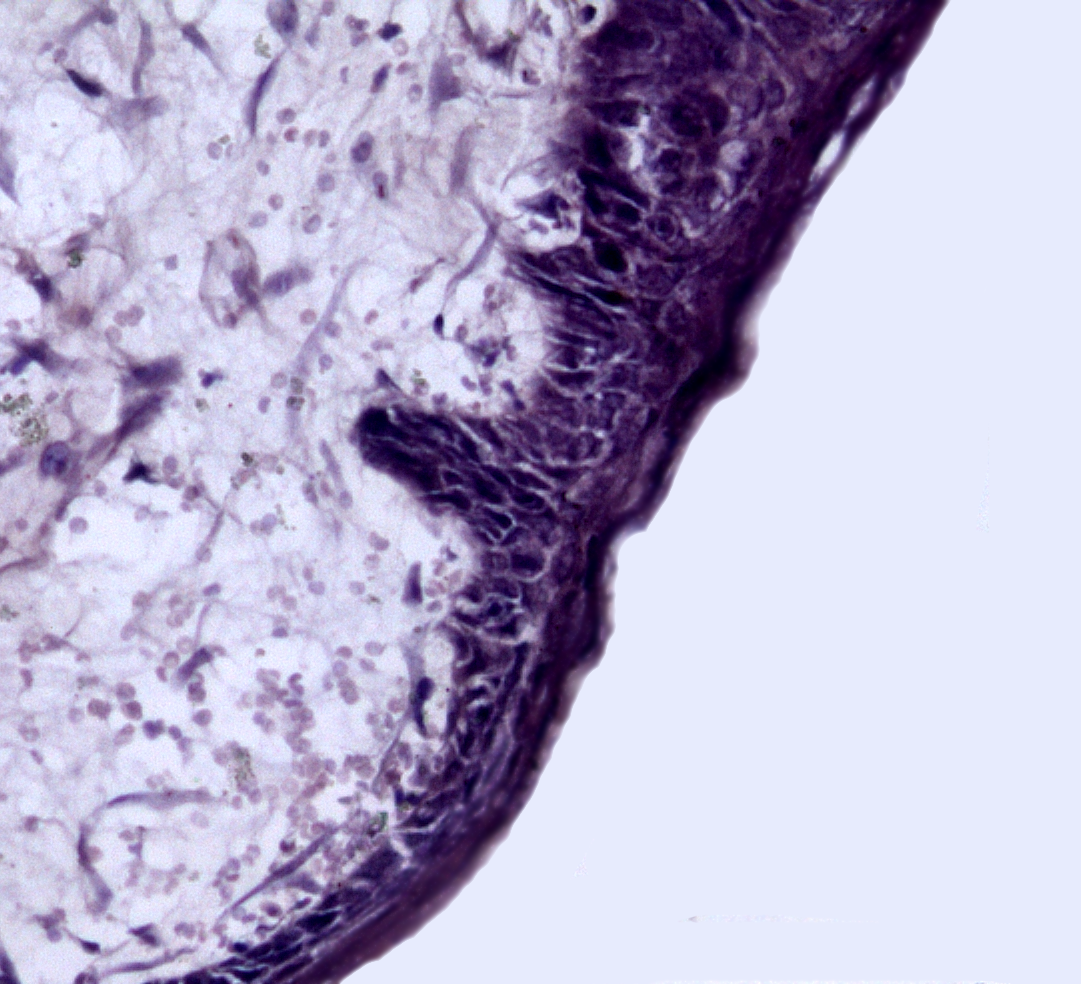


**D**


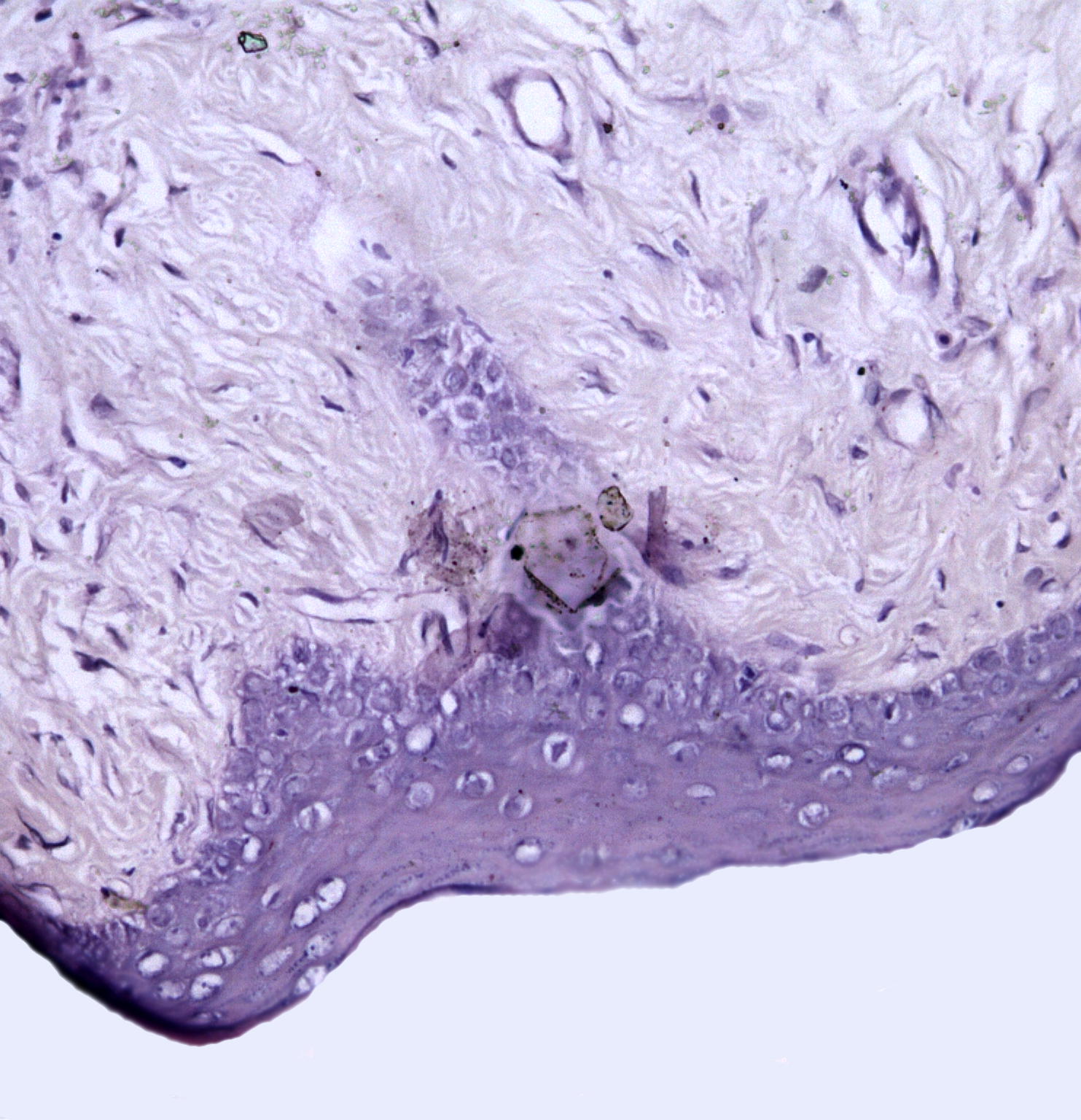


**C**

**Supplementary figure S1.** H&E stained regions of buccal pouch mucosa of control and experimental animals. (20x).

**i**

1. Normal buccal pouch epithelium from control hamster (0 week).
2. Buccal pouch epithelium from a hamster painted with DMBA for 4 weeks exhibiting mild hyperplasia
3. Buccal pouch epithelium from a hamster painted with DMBA for 8 weeks exhibiting varying degrees of hyperplasia and dysplasia.
4. Buccal pouch epithelium from a hamster painted with DMBA for 12 weeks showing well differentiated SCC.
5. Buccal pouch epithelium from a hamster painted with DMBA for 16 weeks exhibiting well differentiated SCC with extensive infiltration into the connective tissue

**ii**

1. Buccal pouch epithelium from control group exhibiting normal intact epithelium.
2. Buccal pouch epithelium from DMBA painted group exhibiting well differentiated SCC with extensive infiltration into connective tissue
3. Buccal pouch epithelium from DMBA+Nimbolide treated group exhibiting low grade tumor.
4. Buccal pouch epithelium from DMBA+Wortmannin treated group exhibiting low grade tumor.
